# Local Immune Responsiveness of Mice Bearing Premalignant Oral Lesions to PD-1 Antibody Treatment

**DOI:** 10.3390/cancers9060062

**Published:** 2017-06-02

**Authors:** Corinne A. Levingston, M. Rita I. Young

**Affiliations:** 1 Research Service, Ralph H. Johnson VA Medical Center, Charleston, SC 29401, USA; cos25@musc.edu; 2 Department of Otolaryngology–Head and Neck Surgery, Medical University of South Carolina, Charleston, SC 29425, USA

**Keywords:** cytokines, head and neck cancer, head and neck squamous cell carcinoma, immunotherapy, PD-1, premalignant oral lesions, T cell

## Abstract

A carcinogen-induced premalignant oral lesion model that progresses to oral cancer was used to examine the immunological impact of a 5-week treatment regimen to block programmed cell death protein 1 (PD-1). PD-1 antibody treatment resulted in concurrent, but transient, increases in interleukin (IL)-2, IFN-γ and IL-17, and delayed increases in IL-6 and IL-10 within the lesion-bearing tongue epithelium. In contrast, cytokine secretion by lymph node cells of PD-1 antibody-treated mice was lower than for mice treated with control antibodies, with the exception of interferon (IFN)-γ, whose secretion increased late in the treatment period. This delayed secretion of IFN-γ coincided with an increase in CD4^+^ lymph node cells expressing IFN-γ. Lymph node cells of PD-1 antibody-treated mice reacted to a challenge with lysates of lesions or cancer by early production of IFN-γ, but this rapidly subsided. There also was increased production IL-17 and tumor necrosis factor (TNF)-α in response to the challenge, but the response was greatest by cells of control lesion-bearing mice. Clinical assessment showed an early but transient, stabilization of disease in mice treated with PD-1 antibody. These results show an early beneficial, but time-limited, response to PD-1 antibody treatment, which then fails with continued lesion progression.

## 1. Introduction

Strategies to block immune checkpoint inhibitors are now being more commonly used as added treatment approaches for cancer patients, with the goal of stimulating anti-tumor immune defenses. For example, several studies have shown clinically meaningful responses following treatment of patients bearing head and neck squamous cell carcinoma (HNSCC) with antibodies to programmed death receptor-1 (PD-1) [1,2]. Clinical effectiveness of anti-PD-1 antibodies was also shown for ovarian cancer [3]. When treatment of renal cell carcinoma patients with PD-1 antibody was continued even after disease progression, a significant proportion of patients showed tumor reduction or disease stabilization [4]. PD-1 antibodies also prolonged progression-free survival of patients with advanced melanoma [5]. In this latter study, treatment with antibodies to PD-1 had increased clinical effectiveness over treatment with antibodies to a different checkpoint, cytotoxic T-lymphocyte-associated protein 4 (CTLA-4). Effectiveness of PD-1 antibodies was also shown for melanoma patients that were refractory to treatment with antibodies to CTLA-4 [6]. By combining treatment with anti-PD-1 and anti-CTLA-4 antibodies, treatment effectiveness for metastatic melanoma patients was shown to be further increased [7]. The ligand PD-L1 has also been targeted in clinical trials. Treatment of patients having non-small-cell lung cancer with a blocking PD-L1 antibody increased their survival over that of control patients that were treated with docetaxel [8]. Some responses were also seen in patients with metastatic renal cell carcinoma [9]. 

There has been recognition of some limitations to treatments aiming to block checkpoint pathways. Development of resistance to PD-1 antibody treatment in melanoma patients and in a mouse lung cancer model has been demonstrated [10,11]. Studies in animal models identified resistance to treatment in part due to upregulation of other checkpoint inhibitors when the PD-1/PD-L1 axis is blocked [12]. This provided support for using combination treatments to block several inhibitors, including PD-L1 and CTLA-4 [13]. Studies with human cancer specimens have associated resistance to PD-1 blockade with mutations in interferon signaling pathways [14]. 

Immunological treatment of cancers is complicated by the armament of immune inhibitory approaches induced by cancers. In addition to the above-mentioned resistance to treatments aiming to block checkpoint pathways, cancers induce other immune inhibitory and cancer-protective mechanisms. These include not only cancer release of soluble immune inhibitory mediators such as prostaglandin E_2_ (PGE_2_), transforming growth factor (TGF)-β and interleukin (IL)-10, but also the induction of host immune inhibitory cells such as M2 macrophages, Treg cells, Th2 skewed T-cells, myeloid-derived suppressor cells (MDSC) and the less mature CD34^+^ progenitor cells [8,15,16,17,18,19,20,21,22]. HNSCC is a malignancy that induces pronounced immune inhibition through several of these mechanisms [23,24,25]. 

What has not been sufficiently tested is immunological treatment against precancerous lesions that have not yet induced extensive immune inhibition, but which are at high risk for secondary occurrences or progressing to cancer. Our prior studies with patient specimens and in a carcinogen-induced murine premalignant oral lesions model had shown that, during progression to cancer, there are initially increased levels of immune activity, including inflammatory and Th1 activity, but a decline in this activity as lesions progress to cancer and once cancer develops [25,26,27]. A few approaches have been tested in the premalignant lesion mouse model to sustain the increased immune activity in premalignant lesions, including cytokine treatment to sustain Th17 cells, indomethacin treatment to prevent PGE_2_-mediated immune inhibition, and blockade of PD-1 inhibitor checkpoint [28,29,30]. In this latter study, spleen cell activity of mice bearing premalignant oral lesions was shown to be increased following onset of treatment with PD-1 antibodies [29]. However, this increase was transient. Not previously analyzed is the immunological impact of PD-1 antibody treatment on the local immune milieu within the premalignant lesion area and regional lymph nodes. The present study examined the cytokine responses in the tongue and regional lymph nodes of mice bearing premalignant oral lesions following treatment with PD-1 blocking antibodies. Also examined was the reactivity of lymph node cells to a challenge with lysates of premalignant lesions and HNSCC. The results showed a more prominent response in the lesion-bearing tongue tissue than in lymph nodes, but this response being transient and differed with the different cytokines. Transient clinical responses were also demonstrated.

## 2. Results

### 2.1. Transient Modulation of Cytokine Levels in Tongue Epithelial Tissues of Premalignant Lesion-Bearing Mice Treated with PD-1 Antibodies 

**Figure 1:**
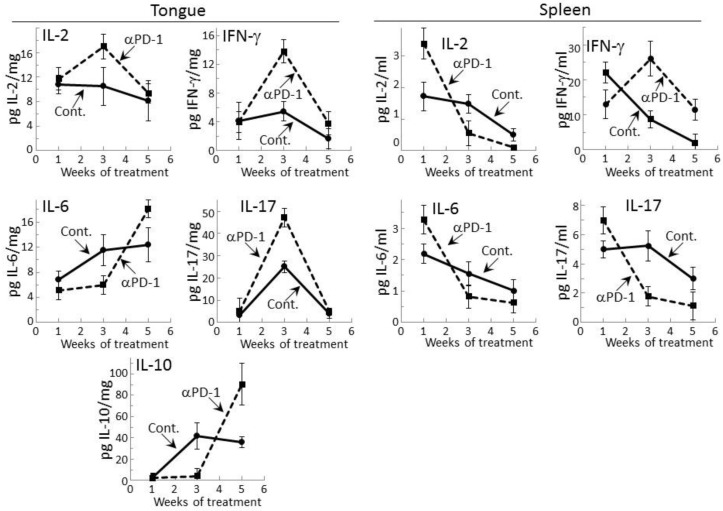
Transient cytokine stimulation in tongue tissue and by spleen cells of premalignant oral lesion-bearing mice treated with programmed cell death (PD)-1 antibody. Mice with carcinogen-induced premalignant oral lesions were sacrificed after 1, 3 and 5 weeks of treatment with isotype control antibodies (Cont.) or PD-1 antibodies (αPD-1). Levels of cytokines interleukin (IL)-2, interferon (IFN)-γ, IL-6, IL-17, and IL-10 in tongue tissue and levels secreted by spleen cells were compared among the treatment groups. Shown are levels of cytokines (mean ± standard error of the mean [SEM]).

Levels of representative Th1, inflammatory and inhibitory cytokines were measured within lysates of premalignant lesion-containing tongue epithelium collected from mice after 1, 3, or 5 weeks of treatment with PD-1 antibody or isotype control antibody (Figure 1). Levels of the Th1 cytokines IL-2 and IFN-γ, the inflammatory cytokines IL-6 and IL-17, and the inhibitory cytokine IL-10 were detectable. The inflammatory mediator tumor necrosis factor (TNF)-α and the inhibitory mediator IL-4 were also measured, but levels in the tongue epithelial lysates were undetectable. After one week of treatment, the tongue epithelium of mice treated with PD-1 or control antibodies contained similar levels of each of the mediators whose levels were detectable. However, after 3 weeks of PD-1 antibody treatment, levels of the Th1 mediators were increased as were levels of the inflammatory mediator IL-17 over levels in tissue lysates of isotype control-treated mice (*p* < 0.02 for IL-2, *p* < 0.002 for IFN-γ and *p* < 0.001 for Th17). At 5 weeks of PD-1 antibody treatment, each of these cytokines whose levels were increased at 3 weeks declined. Levels of the inflammatory mediator IL-6 and the inhibitory mediator IL-10 were reduced in the tongue epithelium of PD-1 antibody-treated mice at 3 weeks (*p* < 0.05 for IL-6 and IL-10), but there was then a delayed increase at 5 weeks compared to levels in isotype control-treated mice (*p* < 0.05 for IL-6 and *p* < 0.005 for IL-10). 

The results of the above studies of cytokine levels in tongue epithelium of mice with premalignant oral lesions differed somewhat from those that we previously showed and currently confirmed for spleen cells [29]. In this comparison, the absolute levels of cytokines could not be equated as the values for tongue epithelium were in pg per mg of lysed epithelial tissue while levels for spleen were pg cytokines secreted per mL. As previously shown, increased levels of IL-2 (*p* < 0.01), IL-6 (*p* < 0.05) and IL-17 (*p* < 0.05) were secreted by spleen cells after one week of PD-1 antibody treatment (Figure 1). Contrasting with the delayed increases in levels of these cytokines in the tongue epithelium, secretion of cytokines by spleen cells of PD-1 antibody-treated mice peaked early at one week and declined thereafter. The exception was spleen cell secretion of IFN-γ which, as we previously showed, peaked at 3 weeks (*p* < 0.01). After 5 weeks of treatment, values for most of the detectable cytokines for both tongue and spleen samples from PD-1 and control antibody-treated mice were declining, with the exception of levels of IL-6 and IL-10 in the tongue. 

### 2.2. PD-1 Antibody Treatment Tempers Cytokine Secretion by Regional Lymph Node Cells of Premalignant Lesion-Bearing Mice

**Figure 2:**
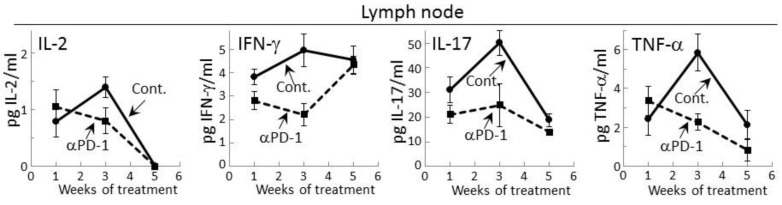
Treatment of premalignant lesion-bearing mice with PD-1 antibody tempers their lymph node cell secretion of most cytokines, except for IFN-γ. Mice with 4NQO-induced premalignant oral lesions were sacrificed after 1, 3 and 5 weeks of treatment with isotype control antibodies or PD-1 antibodies (αPD-1) and secretion of cytokines by their lymph node cells was compared. Shown are levels of secreted cytokines (mean ± SEM).

Prior studies had shown that during the development of premalignant oral lesions, cytokine production by lymph node cells increases, but then declines as the lesions progress to becoming cancers [25,27]. Therefore, cytokine secretion by regional lymph node cells of premalignant lesion-bearing mice that were treated with control or PD-1 antibodies was measured. Detectable levels of IL-2, IFN-γ, IL-17 and TNF-α were secreted by lymph node cells of both control and PD-1 antibody-treated mice (Figure 2). In contrast, secreted levels of IL-6, IL-4 or IL-10 by lymph node cells was not detectable for either group of lesion-bearing mice. During the course of antibody treatment, secretion of each of the cytokines that were detectable was generally lower for lymph node cells of PD-1 antibody-treated mice compared to what was secreted by lymph node cells of control antibody-treated mice (Figure 2). This was seen after one week of PD-1 antibody treatment for secreted levels of IFN-γ (*p* < 0.05) and IL-17 (*p* < 0.05). At week 3, lymph node cells of control mice further increased their secretion of IL-2 (*p* < 0.05), IFN-γ (*p* < 0.05), IL-17 (*p* < 0.01) and TNF-α (*p* < 0.002), but this increase was not seen for lymph node cells of PD-1 antibody-treated mice. By 5 weeks, secretion of IL-2, IL-17 and TNF-α by lymph node cells from both groups of mice declined, with the exception of IFN-γ. At 5 weeks, secretion of IFN-γ by lymph node cells of control mice persisted at the same levels as seen at 3 weeks, but secretion by lymph node cells from PD-1 antibody-treated mice increased (*p* < 0.01) to the levels secreted by lymph node cells from control mice. 

### 2.3. Delayed Increase in Levels of IFN-γ-expressing CD4^+^ cells in Lymph Nodes of PD-1 Antibody-Treated Mice Bearing Premalignant Oral Lesions

**Figure 3:**
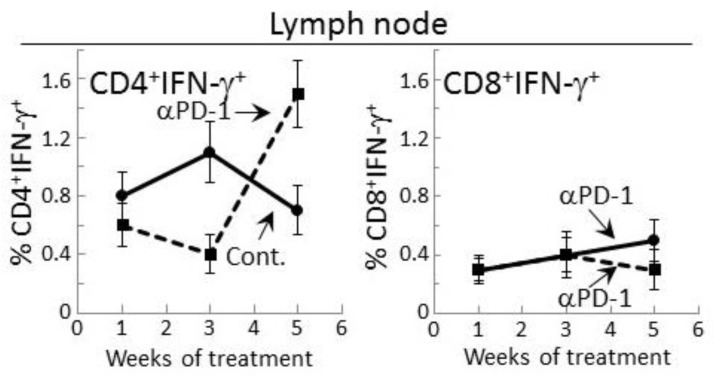
Increase in IFN-γ expression by CD4^+^ lymph node cells from mice bearing premalignant oral lesions after 5 weeks of PD-1 antibody treatment. Mice bearing 4NQO-induced premalignant oral lesions were treated with isotype control antibodies or PD-1 antibodies (αPD-1). After 1, 3 and 5 weeks, groups of mice were sacrificed and their spleens were immunostained extracellularly for CD4 or CD8 and intracellularly for IFN-γ. Shown are percentages of positive-staining cells (mean ± SEM).

The delayed increase in IFN-γ secretion by lymph node cells of PD-1 antibody-treated lesion-bearing mice that was shown above prompted assessment of whether this corresponded with changes in levels of CD4^+^ or CD8^+^ lymph node cells expressing IFN-γ. Consistent with the secreted levels shown in Figure 2, the proportion of CD4^+^ cells expressing IFN-γ among lymph node cells of control lesion-bearing mice was increased at week 3 over levels seen for lesion-bearing mice that were treated with anti-PD-1 antibodies (Figure 3). By week 5 of control antibody treatment, levels of CD4^+^ cells expressing IFN-γ within lymph nodes declined. In contrast, the frequency of IFN-γ-expressing CD4^+^ cells from PD-1 antibody-treated mice increased at week 5 (*p* < 0.01). This delayed increase in IFN-γ-expressing CD4^+^ cells in mice after 5 weeks of anti-PD-1 treatment is consistent with the delayed rise in levels of secreted IFN-γ shown in Figure 2. 

Levels of CD8^+^ cells expressing IFN-γ were also measured among lymph node cells of control or PD-1 antibody-treated lesion-bearing mice (Figure 3). The proportion of these cells was lower than levels of CD4^+^ cells expressing IFN-γ. Contrasting with the changes in levels of CD4^+^ cells expressing IFN-γ, levels of CD8^+^ cells expressing IFN-γ remained relatively constant with only a slight (statistically insignificant) decline among lymph node cells of mice that were treated for 5 weeks with PD-1 antibody.

### 2.4. Transient IFN-γ Responses but Reduced Inflammatory Cytokine Responses by Lymph Node Cells from PD-1 Antibody-Treated Lesion-Bearing Mice to a Challenge of Tongue Premalignant Lesion and Head and Neck Squamous Cell Carcinoma Lysates

**Figure 4:**
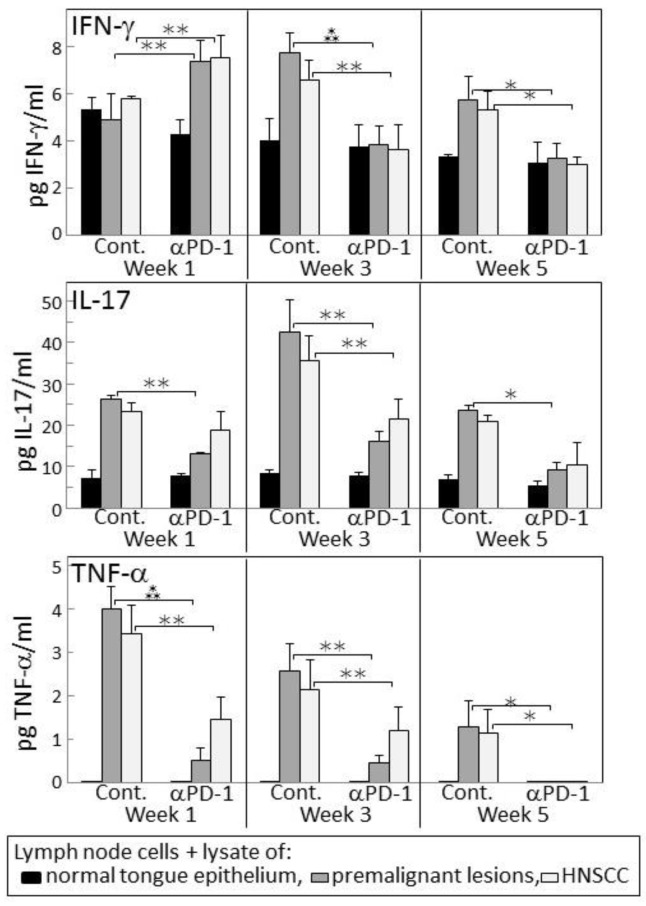
PD-1 antibody treatment of mice bearing premalignant oral lesions transiently increases lymph node cell IFN-γ responses to a challenge of lysates of premalignant lesion or head and neck squamous cell carcinoma (HNSCC) tongue tissues, but not IL-17 or tumor necrosis factor (TNF)-α responses. Mice bearing 4NQO-induced premalignant oral lesions were treated with either isotype control antibodies or PD-1 antibodies (αPD-1). After 1, 3 and 5 weeks, groups of mice were sacrificed and their lymph node cells were cultured with lysates of control tongue tissues or lysates of premalignant lesion or HNSCC tongue tissues. Shown are levels of secreted cytokines by lymph node cells (mean ± SEM). * = *p* < 0.05; ** = *p* < 0.02; *** = *p* < 0.01.

Since 4-nitroquinoline 1-oxide (4NQO)-induced premalignant oral lesions and oral cancer share expression of a number of tumor antigens [31], studies were conducted to determine if treating premalignant lesion-bearing mice with PD-1 antibody would stimulate reactivity of their lymph node cells to an in vitro challenge of premalignant lesions or HNSCC. Also determined was how this reactivity might change as lesions progress toward cancer. Lymph node cells were challenged by culture with lysates of epithelium from control, premalignant lesion or HNSCC tongue tissues. The responsiveness of these lymph node cells to the premalignant lesion or tumor lysates was then measured by their production of IFN-γ, IL-17 and TNF-α (Figure 4). After one week of anti-PD-1 treatment, lymph node cells of the lesion-bearing mice secreted higher levels of IFN-γ in response to premalignant (*p* < 0.001) or HNSCC (*p* < 0.01) challenges compared to their IFN-γ production in response to a challenge of normal tongue epithelium. However, this IFN-γ response to lesion or tumor lysate was transient as it was no longer seen after 3 or 5 weeks of PD-1 antibody treatment. In contrast, as lesions progressed to 3 weeks and 5 weeks following their appearance in control mice, their lymph node cells increased their secretion of IFN-γ in response to a challenge of lesion and HNSCC lysates (*p* < 0.01 at 3 weeks and *p* < 0.05 at 5 weeks).

Lymph node cell production of the inflammatory cytokines IL-17 and TNF-α in response to a challenge of lesion or HNSCC lysates differed from the IFN-γ response (Figure 4). In cultures containing lysate of normal tongue epithelium, basal levels of IL-17 were secreted by lymph node cells of both control and PD-1 antibody-treated mice, but the lymph node cells did not secrete detectable levels of TNF-α (Figure 4). In response to the premalignant lesion or HNSCC challenges, lymph node cells of PD-1 antibody-treated mice produced increased levels of IL-17 and TNF-α at weeks 1 and 3 (both IL-17 and TNF-α responses to premalignant and HNSCC *p* < 0.05 at weeks 1 and 3). However, these responses were less prominent than the responses by lymph node cells of control mice (IL-17 responses to both premalignant and HNSCC: *p* < 0.01 at each of the time points; TNF-α responses to lesion or HNSCC: *p* < 0.01 at weeks 1 and 3, and *p* < 0.05 at week 5). Overall, these results show an early increase in IFN-γ responses by lymph node cells of PD-1 antibody-treated mice to a lesion or tumor challenge, but a diminished inflammatory cytokine response which, instead, is more prominent for lymph node cells of control antibody-treated mice.

**Figure 5:**
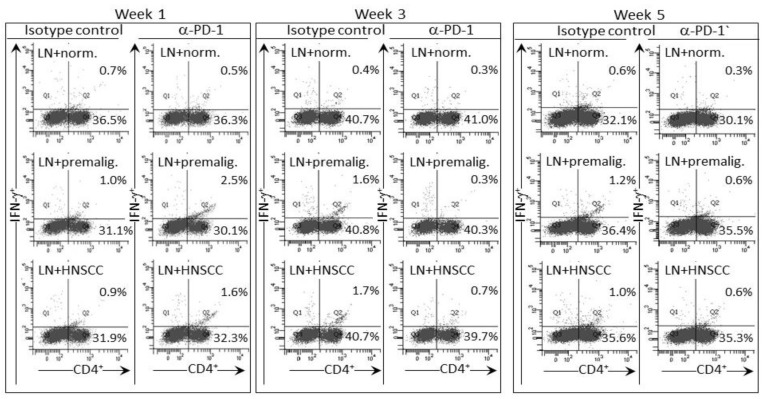
PD-1 antibody treatment of mice bearing premalignant oral lesions transiently increases expression of IFN-γ by lymph node CD4^+^ cells in response to a challenge of lysates of premalignant lesion or HNSCC tongue tissues. Mice bearing 4NQO-induced premalignant oral lesions were treated with either isotype control antibodies or PD-1 antibodies (αPD-1). After 1, 3 and 5 weeks of treatment, sample mice were sacrificed and their lymph node cells (LN) were cultured with lysates of control normal tongue tissue (norm.) or lysates of premalignant lesion (premalig.) or HNSCC tongue tissues. Cultures were then immunostained for CD4 and IFN-γ. Shown are representative fluorescent dot-blots of spleen cells from mice treated with isotype control or anti-PD-1 antibodies.

**Figure 6:**
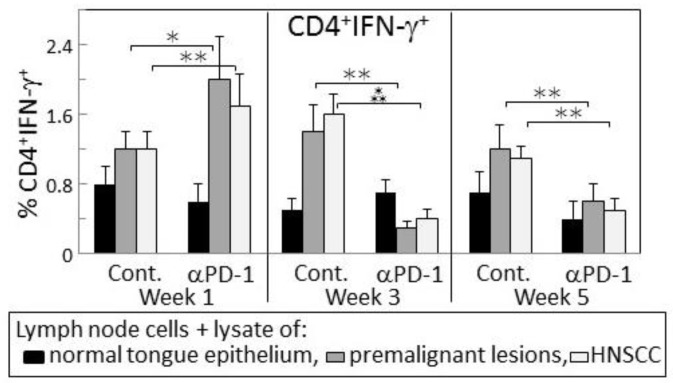
PD-1 antibody treatment of mice bearing premalignant oral lesions transiently increases expression of IFN-γ by lymph node CD4^+^ cells in response to a challenge of lysates of premalignant lesion or HNSCC tongue tissues. Mice bearing 4NQO-induced premalignant oral lesions were treated with either isotype control antibodies or PD-1 antibodies (αPD-1). After 1, 3 and 5 weeks of treatment, sample mice were sacrificed and their lymph node cells were cultured with lysates of control normal tongue tissue or lysates of premalignant lesion or HNSCC tongue tissues. Cultures were then immunostained for CD4 and IFN-γ. Shown are percentages of positive-staining cells (mean ± SEM). * = *p* < 0.05; ** = *p* < 0.02; *** = *p* < 0.01.

The IFN-γ response of lymph node cells of PD-1 antibody-treated mice to a premalignant lesion and HNSCC challenge was also assessed by measuring levels of CD4^+^ cells expressing IFN-γ (examples of cytometric analyses in Figure 5 and summaries in Figure 6). Consistent with the results for IFN-γ secretion, levels of CD4^+^ cells expressing IFN-γ among lymph node cells from mice treated for one week with PD-1 antibody were increased after culture with a premalignant lesion or HNSCC challenge (*p* < 0.05). This responsiveness was significantly greater than that seen for lymph node cells from mice that were treated for 1 week with control antibodies. The response by lymph node cells of PD-1 antibody-treated mice was transient as it was no longer seen for lymph node cells from mice that continued to be treated for 3 or 5 weeks. In contrast, a prominent response to a premalignant lesion or HNSCC challenge was seen for lymph node cells from control antibody-treated mice whose lesions progressed for 3 (*p* < 0.01) and 5 weeks (*p* < 0.05) following lesion detection. Overall, both the phenotypic analyses for IFN-γ-expressing cells and the analyses of IFN-γ secretion show responsiveness to challenges of premalignant lesion and HNSCC lysates by lymph node cells of mice treated for one week with PD-1 antibody, but a waning of this responsiveness with continued PD-1 antibody treatment. Instead, the lymph node cells of control antibody-treated mice mount an IFN-γ response to challenge transiently during later periods, but this also starts to wane with further lesion progression. 

### 2.5. PD-1 Antibody Treatment Transiently Stabilizes Lesion Progression Toward Head and Neck Squamous Cell 

**Figure 7:**
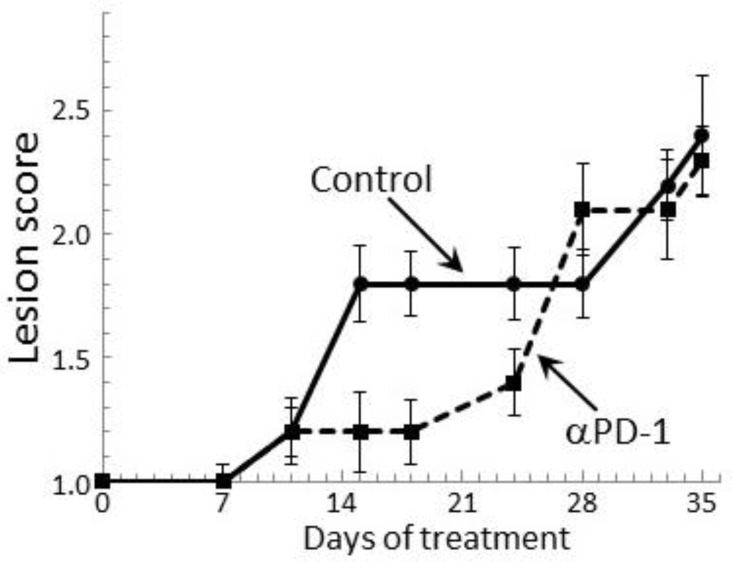
Transient clinical response of mice bearing premalignant oral lesions to treatment with PD-1 antibodies. Mice with premalignant oral lesions were divided into groups of 15 mice each and initiated on treatment with isotype control antibodies or PD-1 antibodies (αPD-1). Progression of lesions toward cancer development was monitored by endoscopic examination of the oral cavity. These endoscopic images were quantitated in a blinded manner by counting the number of visible lesions, and giving lesions a gross pathologic score between 1 and 4 based on their horizontal and vertical size, and overall appearance. The following criteria were used for scoring: 1: flat macule, 2: raised papule, 3: raised plaque, 4: grossly exophytic. Shown are means ± SEM of clinical analyses.

Our prior study showed a transient delay in the progression of premalignant oral lesions toward cancer in mice that were treated with PD-1 antibody, but then a pronounce advancement toward cancer [29]. This clinical response was confirmed in the present study by endoscopic examination of the oral cavity (Figure 7). At 2 weeks of antibody treatment, the oral lesions of PD-1 antibody-treated mice stabilized, while the lesions of control antibody-treated mice progressed in severity. Ten days after the initial lesion stabilization in PD-1 antibody-treated mice, lesions still appeared relatively stable, although there was an increase in lesion severity on some of the mice (*p* < 0.05 on days 15, 18 and 24). However, by 4 weeks of treatment, lesions of PD-1 antibody-treated mice rapidly advanced to similar stages as for control-treated mice and they remained similar between the two groups until mice were euthanized. Thus, despite early stabilization of disease progression in PD-1 antibody-treated mice, the lesions subsequently progressed similar to the progression in control mice.

## 3. Discussion

While immunotherapy to interrupt the PD-1/PD-L1 axis has been used for a variety of cancers, it has not been tested as a strategy to prevent progression of premalignant lesions to cancer. Our prior studies with tissue from patients and with a carcinogen-induced premalignant oral lesion model that progresses to cancer showed increases in Th1 and inflammatory cytokines at the time that premalignant lesions are present, but these increases decline with cancer development [27,32]. Using the carcinogen-induced premalignant oral lesion model we previously showed that treatment with anti-PD-1 antibody resulted in a rapid, but very transient increase in spleen cell cytokine expression and resulted in a transient delay in lesion progression to cancer [29]. However, studies have not been previously conducted to determine the immunological impact of anti-PD-1 antibody at the local level: within the oral premalignant tongue lesions or within regional lymph nodes of mice bearing oral premalignant lesions. This was assessed in the present study, which showed that within the lesion-containing tongue epithelium, levels of the Th1 cytokines IL-2 and IFN-γ, and the inflammatory mediator IL-17 increased in mice treated for 3 weeks with PD-1 antibodies compared to levels in tongue epithelium of isotype control-treated mice. However, by 5 weeks of treatment, levels of each of these cytokines declined. Levels of the inflammatory mediator IL-6 also increased, but this increase was delayed until 5 weeks of treatment. 

The present study also confirmed our prior results demonstrating that PD-1 treatment caused an early, but transient, increase in spleen cell production of Th1 and inflammatory cytokines which was seen after 1 week of PD-1 antibody treatment and declined thereafter [29]. The exception was IFN-γ, which peaked after 3 weeks of treatment before declining. This allowed a comparison of the kinetics. This comparison in the present study showed a delayed cytokine response within the lesion-containing tongue tissue compared to cytokine responses in the spleen. Similar to what was seen in the spleen, the increase in cytokine levels in tongue tissue of mice receiving PD-1 antibody treatment was transient. 

Cytokine production by regional lymph node cells differed from what was seen for the spleen and tongue in that PD-1 antibody treatment tempered cytokine responses, with the exception of delayed stimulation of IFN-γ production. Such tempering of reactivity, including inflammatory responses, was unexpected as interrupting the checkpoint signals would be expected to increase immune activity. Production of IFN-γ was the exception in that levels of IFN-γ produced by lymph node cells after 1 and 3 weeks of anti-PD-1 treatment were lower than those produced by lymph node cells after control antibody treatment, but then increased to levels produced by lymph node cells of the control treatment group at week 5. The present study also showed there was a delayed increase in levels of CD4^+^ cells that expressed IFN-γ within the lymph nodes of anti-PD-1 antibody-treated mice. Thus, it may be that lymph node secretion of IFN-γ might have continued to increase after 5 weeks of treatment if the duration of the study were prolonged. 

The transiency of the boost in cytokine levels in both the spleen and tongue tissue is consistent with demonstrations of acquired resistance to PD-1 antibody treatment in melanoma patients and in a mouse lung cancer model [10,11]. This development of resistance in melanoma patients and in the lung cancer model was shown to be associated with alterations in interferon receptor signaling. A separate study with a lung cancer mouse model and with cancer patients demonstrated that resistance to PD-1 blockade was associated with upregulation of other immune checkpoints [12]. Whether or not the transiency of the PD-1 antibody-associated increases in cytokine levels in the spleen and tongue in our premalignant oral lesion model is due to shifts to other immune checkpoints or due to interference in IFN-γ signaling has not yet been determined. What we had previously demonstrated was an increase in production of the inhibitory mediator PGE_2_ by premalignant lesion cells at time points similar to the last time point assessed in the present study [30]. Furthermore, this latter study had also shown that treatment of lesion-bearing mice with indomethacin to block prostaglandin production increased production of IFN-γ. Thus, it may be that the transient nature of the increase in cytokines due to PD-1 antibody treatment could be due to development of immune inhibitory mechanisms other than through the PD-1 immune checkpoint axis. This concept is supported by other studies showing only modest clinical effectiveness of checkpoint blockade treatment in a prostate cancer model, but a robust synergistic response when checkpoint blockade treatment was combined with kinase-inhibitory treatment targeting infiltration by myeloid-derived suppressor cells [33]. A separate study also demonstrated alternative inhibitory mechanisms, such as IL-17 stimulation of intratumoral neutrophil infiltration, as contributors to PD-1 blockade resistance by lung cancer [34].

The reactivity of lymph node cells to lysates of premalignant lesion or tongue cancer tissues as compared to normal tongue epithelium was determined by assessing their secretion of cytokines. Measurable levels of IFN-γ, IL-17 and TNF-α were produced in these challenge cultures, while levels of IL-2, IL-6, IL-4 and IL-10 were not detectable. Lymph node cells of mice treated for 1 week with PD-1 antibodies showed increased IFN-γ responses to premalignant lesion or cancer lysates compared to the absence of responsiveness by lymph node cells of control mice. This was seen in both measurements of secreted IFN-γ as well as levels of CD4^+^ cells expressing IFN-γ. These results for IFN-γ contrasted with results for lymph node secretion of the inflammatory mediators IL-17 or TNF-α in response to a lesion or cancer challenge, which was greater for control antibody-treated mice. The responses of lymph node cells to lesion or HNSCC lysate challenges were transient, which is consistent with what we previously demonstrated for spleen cell responses to challenge [29]. It is also consistent with the transient increase in levels of cytokines within tongue tissue of PD-1 antibody-treated mice and with the transient increase in spontaneous spleen cell secretion of cytokines. 

It is interesting and not yet possible to explain why the timing of peak cytokine levels differed among the various tissues analyzed. What is consistent is that, as PD-1 antibody treatment continued, expression of most cytokines declined in all tissues assessed. This is somewhat consistent with this study’s demonstration of an early clinical response to PD-1 antibody treatment, which was not sustained. It is not possible to determine whether the decline in cytokines in mice receiving PD-1 antibody treatment is due to the treatment or due to the progression of premalignant oral lesions to cancer. It can be speculated that, since lesions progressed in both control and PD-1 antibody-treated mice, progression of lesions toward cancer contributed to the loss of PD-1 antibody treatment effectiveness at stimulating immune reactivity. Whether or not this transient effectiveness to stimulate immune reactivity by PD-1 antibody treatment of mice bearing premalignant oral lesions is unique to lesions of the oral cavity or whether the response would be transient in other premalignant lesion models is not know as it has not been tested in other premalignant lesions conditions. Important future studies are needed to determine if even the transient positive immune response resulting from blocking the PD-1 checkpoint could open a window of opportunity to increase effectiveness of immune stimulatory treatments such as tumor vaccines or various adoptive immune cell transfer approaches.

## 4. Materials and Methods 

### 4.1. Oral Premalignant Lesion Model and Treatment Schedule

All studies were carried out in accordance with National Institutes of Health Guide for the Care and Use of Laboratory Animals and were approved by the Institutional Animal Care and Use Committee of record. Female C57BL/6 mice (Charles Rivers Laboratory, Wilmington, MA, USA) were treated with 50 μg/mL 4NQO in their drinking water starting at 2 months of age until development of premalignant oral lesions [32,35]. In this model, premalignant lesions develop on the tongue and their development was monitored by endoscopic examination of the oral cavity. This was accomplished by sedating mice with inhaled isoflurane (Piramal Healthcare, Bethlehem, PA, USA) and examining the oral cavities with a Stryker 1.9 mm × 30° endoscope and a Stryker 1088 HD camera (Stryker, Kalamazoo, MI, USA). The identity of these premalignant oral lesions and HNSCC was confirmed histologically. In studies to determine the clinical effectiveness of treatments, mice were examined by endoscopy twice per week and severity of their lesions was quantitated in a blinded manner. Lesions were given a gross pathologic score between 1 and 4 based on their horizontal and vertical size, and overall appearance [25] The criteria used for scoring included the following: 1: flat macule, 2: raised papule, 3: raised plaque, 4: grossly exophytic. 

### 4.2. Antibody Treatment of Mice Bearing Premalignant Oral Lesions

Treatment with IgG1 isotype control antibodies or anti-PD-1 blocking antibodies (muDX400, Merck & Co., Kenilworth, NJ, USA) was initiated once the tongue premalignant oral lesions became detectable by endoscopy. Mice were treated by intraperitoneal injection of 200 μg of these mouse antibodies at 4-day intervals. After 1, 3 and 5 weeks of treatment, subgroups of mice were sacrificed and their spleens, tongue tissues and regional lymph nodes were collected for immunological analyses.

### 4.3. Tissue Culture Medium

The tissue culture medium used was 1× DMEM (Life Technologies, Grand Island, NY, USA) containing 4.5 g/L d-glucose and l-glutamine. This medium was supplemented with 10% fetal bovine serum and 1× antibiotic antimycotic solution containing penicillin, streptomycin and amphotericin B (Sigma-Aldrich, St. Louis, MO, USA).

### 4.4. Immune Analyses

Levels of cytokines in the premalignant lesion-containing tongue tissue were measured after lysis by sonication. Spleen cells and lymph node cells were used to measure their basal (unstimulated) cytokine secretion. Lymph node cells were also used to measure cytokine secretion in response to a challenge with control lysates of normal tongue tissues or lysates of premalignant oral lesion or HNSCC tongue tissues. In addition, aliquots of lymph node cells were immunostained for expression of IFN-γ by CD4^+^ and CD8^+^ at the time of spleen cell collection and after the lysate challenges. 

For measurement of basal secretion of cytokines, spleen and lymph node cells of control- and anti-PD-1-treated mice bearing premalignant oral lesions were seeded on anti-CD3-coated tissue culture wells at a density of 1 × 10^6^/mL. After 24 hours of culture, supernatants were collected for measurement of cytokine levels. Studies to assess lymph node cell responsiveness to a challenge of premalignant lesions or HNSCC lysates, cells from control and PD-1 antibody-treated mice were seeded together with lysates of normal tongue epithelium, premalignant lesion or HNSCC tissues into anti-CD3-coated tissue culture plates. After 3 days of culture, supernatants were collected and used for cytokine measurement and cells were collected for immunostaining. Lysates for the challenges were prepared by stripping the epithelial layers of tongue tissues through enzymatic digestion at 37 °C with 0.23 U Liberase (Sigma-Aldrich) for 4 h. The dissociated tissues were then washed, lysed by sonication, and protein levels were equalized. At the levels that they were added to the lymph node cultures, the lysate challenges had undetectable levels of cytokines. 

### 4.5. Cytokine Bead Array

The levels of IFN-γ, IL-2, IL-17A, IL-4, IL-6 and IL-10 in cell culture supernatants were determined using a mouse cytometric bead array (CBA) Th1/Th2/Th17 cytokine kits (BD Biosciences, San Jose, CA, USA). Cytokine profiles were measured with a FACS Canto (BD Biosciences) flow cytometer and relative amounts were calculated using FCAP Array Software version 3.0 (manufactured by Soft Flow Hungary Ltd. (St. Louis Park, MN, USA) for BD Biosciences). 

### 4.6. Phenotypic Analysis of Lymph Node Cells for Expression of IFN-γ

To measure expression of IFN-γ by CD4^+^ and CD8^+^ lymph node cells, they were first treated for 4 h with brefeldin A solution (BD Bioscience) to prevent cytokine secretion. Nonspecific staining of cells was blocked with FBS and anti-CD16/32 monoclonal antibodies. Cells were then surface stained with PerCP-Cy5.5 CD4 and PE-Cy7 CD8a antibodies, fixed and permeabilized with Cytofix/Cytoperm. They were then intracellularly stained with FITC IFN-γ (BD Bioscience). The frequency of CD4^+^ and CD8^+^ cells that stained positive for IFN-γ was measured by flow cytometry (FACSCanto, BD Bioscience).

### 4.7. Statistical Analysis

Data are reported as means ± standard error of the mean. The immunological analyses on cells from control and PD-1 antibody treated mice were conducted in duplicate with 5 mice being analyzed per group at each time point, with all mice being assayed individually. The Mann-Whitney U test was used to determine significance of differences in values between each of two parameters (GraphPad Prism version 6.03 for Windows, GraphPad Software, La Jolla, CA, USA). Significance was reported in the 95% confidence interval.
